# A Comparative Study of* Actinidia deliciosa* and* Garcinia mangostana* in Ovariectomy-Induced Osteoporosis in Female Wistar Rats

**DOI:** 10.1155/2017/5349520

**Published:** 2017-12-13

**Authors:** Chitra Vellapandian, Evelyn Sharon Sukumaran, Logeshwaran Ramalingam Sivasubramanian, Venkataramanan Rajabatar Vetrivelan

**Affiliations:** Department of Pharmacology, SRM College of Pharmacy, SRM Institute of Science and Technology (Formerly SRM University), Chennai, India

## Abstract

The present study was designed to evaluate antiosteoporotic activity of the fresh juice mixtures obtained from* Actinidia deliciosa* and* Garcinia mangostana* as well as the pericarp extract of* Garcinia mangostana *on postmenopausal osteoporosis. 3-month-old female Wistar rats were ovariectiomized and the treatment began 14 days after ovariectomy and continued for 40 days. Statistically significant changes were noticed in body weight, ash weight, bone mineral content, and femur length and weight followed by serum evaluation and histopathology of femur bone. Administration of the fresh juice mixtures of the fruits of* Actinidia deliciosa* and* Garcinia mangostana* prevented ovariectomy-induced bone loss. The administration of the fresh juice mixtures resulted in an increase in the femur length and weight, followed by an increase in the body weight as well as the calcium content obtained from the ash of the femur bone. It is evident that the fresh juice mixtures can be used as a remedy as well as a prophylactic for the prevention of postmenopausal osteoporosis. The present study showed that the combined effect of the fruit juice mixtures of* Actinidia deliciosa* and* Garcinia mangostana* was found to be a better treatment for postmenopausal osteoporosis when compared to the pericarp extract of* Garcinia mangostana*.

## 1. Introduction

Osteoporosis has become a major public threat globally affecting men and women equally. Osteoporosis, as defined by the world health organization, is a progressive skeletal disease characterized by low bone mass and microarchitectural destruction of the bone tissue leading to bone fragility accompanied by increase in fracture risk [1]. Due to an increased longevity of life in India osteoporotic fractures are becoming a major cause of morbidity and mortality similar to that of Western population. It is currently estimated that more than 36 million Indians are affected by osteoporosis [2]. Osteoporosis that is associated with ovarian hormone deficiency following menopause is the most common age related bone loss. Postmenopausal osteoporosis (Type 1) is the most common type in women after menopause linked to an estrogen deficiency [3]. During the menopausal transition women experience a number of problems like hot flashes, night sweats, vaginal dryness, and so on. Besides these, postmenopausal osteoporosis has become the most prevalent disease among women [4]. In recent times, the proportion of menopausal women is rising due to the expanding aging population. Thus the health of menopausal women is becoming a primary concern worldwide [4]. Many synthetic drugs including raloxifene, droloxifene, bisphosphonates, calcitonin, and hormonal replacement therapy have been used to treat postmenopausal osteoporosis. But their long-term use was found to bring many side effects such as increased risk of endometrial and breast cancer, vaginal bleeding, hot flashes, and breast tenderness [5, 6]. Thus it is absolutely essential to find naturally occurring substances which have greater influence to novate the current hormonal replacement therapy due to its high cost and to bypass the health hazard associated with it.

*Actinidia deliciosa* and* Garcinia mangostana* were the chosen fruits for the present study. These fruits have become a popular dietary supplement in recent times due to their potential health promoting benefits.* Actinidia deliciosa *popularly known as kiwi or the Chinese gooseberry has a natural source of carotenoids like provitamin A beta-carotene, lutein, and zeaxanthin followed by rich sources of vitamin C along with vitamins A and E and a considerable amount of potassium [7]. Kiwi is reported to be a remedy for acute burn wounds [8].

*Garcinia mangostana* popularly known as mangustan or mangosteen is a tropical tree grown in south east Asian countries like India, Myanmar, Malaysia, Sri Lanka, and Thailand [9]. The fruit is also called “queen of fruits” [10]. The phytochemical evaluation on* Garcinia mangostana* revealed that it is rich in a variety of oxygenated and prenylated xanthones [11].* Garcinia mangostana* is reported to have neuroprotective [12], antimalarial and toxicity [13], antioxidant [14], hepatocellular carcinoma [15], breast and prostate cancer [16], and anti-inflammatory activity [17, 18].

The effects of* Actinidia deliciosa* and* Garcinia mangostana* on bone health have not been studied so far. Thus this study was aimed at examining the role of fresh juice mixtures of the fruits of* Actinidia deliciosa* and* Garcinia mangostana* as well as the pericarp extract of* Garcinia mangostana* in preventing the osteoporotic changes using ovariectomy-induced osteoporotic model.

## 2. Materials and Methods

### 2.1. Fruit Collection and Authentication

The fresh fruits of* Actinidia deliciosa* and* Garcinia mangostana* were collected from a fruit market in Koyambedu, Chennai. The fresh fruits were authenticated by Professor P. Jayaraman, Ph.D., Director, Plant Anatomy and Research Centre, Tambaram.

### 2.2. Preparation of the Pericarp Extract:* Garcinia mangostana*

The pericarp of* Garcinia mangostana* was shade dried and powdered coarsely and passed through mesh number of size 44. The measurable amount of the powder was defatted with petroleum ether and then extracted with hydroalcohol (70%) at room temperature for 72 hours using Soxhlet apparatus. The extract was then filtered and concentrated in rotary vacuum evaporator and dried in desiccator. 500 gm of the extract was used and the yield of the extraction was 12.7%.

### 2.3. Preparation of the Fresh Fruit Juice Mixtures:* Actinidia deliciosa *and* Garcinia mangostana*

The fresh fruits were washed thoroughly in cold water exfoliating the skin. The flesh was then scooped out and blended in a blender. The liquid was filtered and used.

### 2.4. Animals

Twenty-four three-month-old virgin female Wistar rats weighing 250–300 g were obtained from King Institute, Guindy. The animals were housed under controlled conditions including a room temperature of 22 ± 1°C with 12 : 12-hour light-dark cycle. The animals were fed with standard food pellets and water ad libitum. The procedures of the animal study including the raising, feeding, and surgical procedure were conducted in accordance with CPCSEA guidelines and were approved by IAEC (IAEC number* IAEC/160/2015*).

### 2.5. Experimental Design: Induction of Osteoporosis

Osteoporosis was induced by ovariectomy. Ovariectomisation is the removal of the ovaries. One of the well-established animal models to study osteoporosis is the rat ovariectomy model [19]. The ovariectomised rat is an excellent preclinical animal model that rightly imitates the clinical feature of estrogen depleted human skeleton [19, 20]. The animals were divided into four groups.

**Figure 1:**
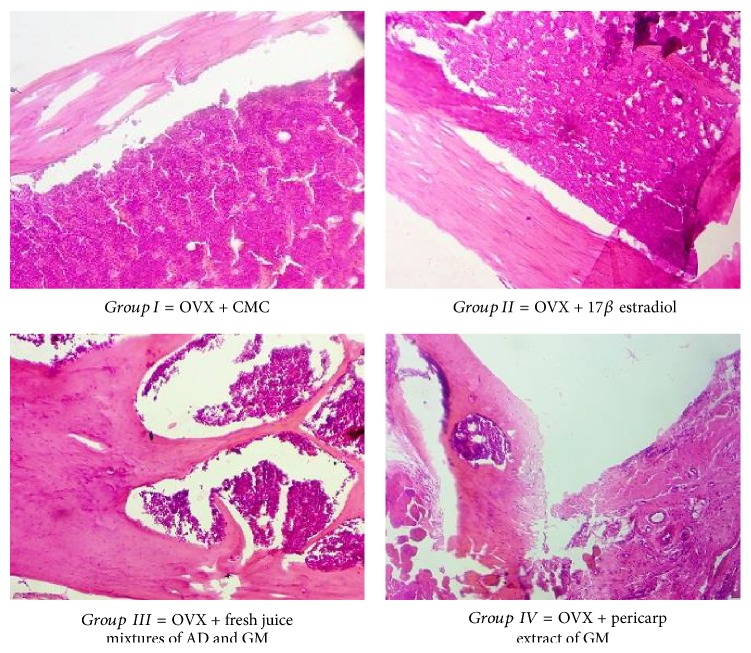
*Group 1* (ovariectomised): dispersion, loss, and thinning of the intertrabecular spaces are evident.* Group 2* (drug treated): sparse femoral region with loss of interconnectivity was noted.* Group 3* (fresh juice treated): trabecular bone with normal architecture is restored with an increase in bone cells.* Group 4* (pericarp extract treated): compact and normal cells are noticed.

Bilateral ovariectomy was performed using cocktail anesthesia of ketamine and xylazine (10 mg/kg). Ovariectomy was done by making a small incision of 1 cm in the dorsal side. The ovary surrounded by fat was identified and the blood vessels supplying the ovary were ligated and the ovary was cut off. The wound was closed using absorbable sutures. Povidone iodine solution was sprayed on the wound. Immediately after surgery tramadol (5 mg/kg) was given intraperitoneally to reduce the postoperative pain [21, 22]. The animals were kept in individual cages for a week after which they were divided into 4 groups (Figure 1).

  Group I: OVX + carboxymethyl cellulose (1 ml/kg) given orally
  Group II: OVX + 17*β* estradiol (10 *μ*g/kg) given orally
  Group III: OVX + fresh juice mixtures of* Actinidia deliciosa* and* Garcinia mangostana* (1 ml/kg) given orally
  Group IV: OVX + pericarp extract of* Garcinia mangostana* (300 *μ*g/kg) given orally.

### 2.6. Experimental Procedure

Treatment was started after 4 weeks of ovariectomisation and continued for 40 days. Body weights were taken at weekly intervals throughout the study period. After 40 days of the treatment blood samples were withdrawn by retroorbital method. Blood samples were allowed to clot at room temperature and the serum was separated by centrifugation at 1000 rpm for 20 min. Serum samples were stored at −20°C until analysis.

The left and the right femur bones were dissected out. The femur bones were thawed, autoclaved for 15 min at 110°C, and divested of the soft tissue for the measurement of femur weight and length. After the length and weight measurement the femur bones were kept in tared silica crucibles and kept in muffle furnace at constant temperature for 24 hours at 1000°C for the determination of ash weight, ash percentage, and the calcium content present in the bone. Histopathology was carried out using the right femur bone [23].

### 2.7. Measurement of Femur Length and Weight

Freshly isolated femur was used for the measurement of its length and weight. The length was measured using digital Vernier callipers. The femur weight was measured using digital weighing balance [24–26].

### 2.8. Measurement of Ash Value, Ash Percentage, and Bone Mineral Density, Calcium

After the measurement of bone length and weight, the bone was kept in tared fused silica crucibles and kept in a muffle furnace and dried to a constant temperature at 1000°C for 24 hours. Then the total ash was weighed and the percentage of ash was calculated from the initial weight of the femur. The bone ash was diluted with deionized water to determine the calcium content present [27–29].

### 2.9. Biomechanical Testing: 3-Point Bending Test

Three-point bending test was performed in the right femora of the ovariectomised rats. The bending test was carried out to analyze the maximum flection load. The isolated femur was placed horizontally between two supports and a load was applied in the middle of the bone at a distortion rate of 2 mm/min until fracture occurred. Stiffness and energy reflecting the structural properties of the bone were calculated by automated computation [6, 30–32].

### 2.10. Biochemical Estimation, Serum

The test was carried out using diagnostic reagent kit for the* in vitro *determination of serum calcium. The calcium present in the serum was precipitated with naphthyl hydroxamic acid (calcium reagent). The precipitate was then dissolved in EDTA reagent and calcium from this solution was complexed with color reagent to give colored complex which was measured colorimetrically [33, 34].

*In vitro *determination of ALP was carried out by using diagnostic reagent kit. ALP from serum converts phenyl phosphate to inorganic phosphate and phenol at pH 10. Phenol so formed in alkaline medium with 4-aminoantipyrine in the presence of oxidizing agent potassium ferricyanide and forms an orange colored complex, which was measured colorimetrically using an autoanalyzer [35–38].

*In vitro *determination of tartrate resistant acid and phosphate was carried out using diagnostic reagent kits. Acid phosphatase from the serum will convert phenyl phosphate to inorganic phosphate and phenol at pH 4.8. Phenol formed will react with aminoantipyrine in the presence of oxidizing agent potassium ferricyanide and will form an orange colored complex, which was measured calorimetrically [5, 6, 39].

### 2.11. Histopathology of the Femur Bone

After the biomechanical testing the femur bones were used for histopathological studies. The femur was fixed in 10% neutral formalin for 12 hours at 4°C and decalcified in 5% EDTA for 7 days followed by embedding in paraffin and cut into 5 *μ*m thickness longitudinally. The section was stained with hematoxylin, eosin, and TRAP (tartrate resistant acid phosphate), a cytochemical marker to find out the osteoclast activity and finally counterstained with hematoxylin. The number of positively stained osteoclasts in the middle section of the femora was enumerated for the groups [23, 40, 41].

### 2.12. Statistical Analysis

All the data obtained in the study was expressed as median ± interquartile range. The comparison was done by Kruskal-Wallis one-way analysis of variance on ranks.

## 3. Results

### 3.1. Body Weight Changes

Table 1 shows the body weight changes in the ovariectomised groups. A maximum weight gain was observed in the fresh juice treated group. There was a comparative decrease in the body weights in all the other treatment groups.

The differences in the median values among the treatment groups are greater than what would be expected by chance; there is a statistically significant difference (*P* = 0.035):

 
*Normality test (Shapiro-Wilk) Week 1: passed (P = 0.197)*
 
*Normality test (Shapiro-Wilk) Week 2: passed (P = 0.230)*
 
*Normality test (Shapiro-Wilk) Week 3: passed (P = 0.179)*
 
*Normality test (Shapiro-Wilk) Week 4: passed (P = 0.394)*
 
*Normality test (Shapiro-Wilk) Week 5: passed (P = 0.392).*

### 3.2. Ash Weight and Bone Mineral Content

Tables 2 and 3 show the ash weight, ash percentage, and calcium content on the isolated femur bone. Fresh juice treated group showed a drastic increase in the ash weight and the calcium levels when compared to the other groups. Treatment prevented the reduction of ash, ash percentage, and the calcium content to an appropriate level determining its usefulness in the prevention of bone loss.

### 3.3. Measurement of Femur Parameter

Ovariectomy resulted in a significant reduction in the femoral length and weight when compared to the other treatment groups. Treatment with the fresh juice mixtures increased the length and weight significantly. Tables 4 and 5 show the effect of the treatment on the femur length and weight.

### 3.4. Biomechanical Analysis: 3-Point Bending Test

Table 6 shows the biomechanical analysis done on the isolated femur bone. It was observed that energy required to break the femur bone of the fresh juice administered group significantly increased when compared to the other drug treated groups.

### 3.5. Biochemical Estimation, Serum

Serum calcium levels were significantly decreased in the control group when compared to the other treated groups. The pericarp treated group showed a significant increase in the serum calcium level when compared to the other groups.

Serum TRAP, the bone marker for the osteoclastic activity, was significantly in the control group. However, when compared to the control group, TRAP level was considerably decreased in all the treated groups.

The ovariectomised control group showed a significant increase in the alkaline phosphatase levels. ALP levels were found to be decreased in the estrogen and pericarp treated groups. There was an equal increase in the ALP levels in the fresh juice treated group similar to that of the control.

Tables 7, 8, and 9 show the effect of treatment of the serum biochemical markers in the ovariectomised rats.

### 3.6. Histopathology of Femur Bone

Histopathological results of the femoral tissue in (A) control, (B) drug treated, (C) fresh juice treated, and (D) pericarp extract treated group were shown using hematoxylin and eosin staining (magnification, ×100). The histopathological study showed that the tissues from the OVX + CMC treated group were thinly scattered with wider intertrabecular spaces.

The ovariectomised group also showed porous perforated and disintegrated bone architecture compared with the groups. Treatment with estrogen and fresh juice showed complete restoration of osteoporosis bone changes to a compact firm. The treated groups showed significant changes of the restoration of the morphological changes (pore formation, disintegrated bone architecture, and reduced compactness) compared with the ovariectomised control rats. Microscopic examination of the femur of the control group showed disruptive and lytic changes and fibrocartilaginous matrix with osteodystrophy. Group 3 and 4 showed significant restorative progress with mineralization along with fairly well-distributed osteocytes.

## 4. Discussion

Osteoporosis a silently progressing metabolic bone disease is widely prevalent in India leading to an increased fracture risk equally in both men and women [42]. The main cause of osteoporosis in women is due to the hormonal changes due to decrease in the estrogen levels in the blood [43]. Postmenopausal osteoporosis in women is often named as a silent disease which leads to decreased bone strength eventually leading to an increased fracture risk [44]. Postmenopausal osteoporosis is caused by accelerated bone resorption and a systemic calcium imbalance resulting from estrogen deficiency induced by menopause [45]. The most frequently used antiosteoporotic drugs developed in affluent countries are expensive for common people in developing and in developed countries. With its numerous clinical complications there is an urgent need to take necessary steps to create awareness and provide a better treatment for postmenopausal osteoporosis. And thus there is an alarming need to manage osteoporosis especially in postmenopausal women. Hence this present study was carried out with an objective to evaluate he osteoprotective effect of* Actinidia deliciosa* and* Garcinia mangostana*.

The commonly used animal model for osteoporosis is the ovariectomised rat. American FDA recommends ovariectomised animals as the preferred model for bone loss research [46]. The ovariectomised rat model exhibits most of the characteristics similar to that of the human postmenopausal osteoporosis. Rats do not experience a natural menopause with ovariectomy procedure; it has become a time honoured method to produce artificial menopause [47]. The ovariectomised rat model has been established because of its ability to stimulate various clinical human syndromes deriving from osteoporosis [48].

There was an increase in the body weight of the ovariectomised rat which might be due to estrogen deficiency [49]. This gain in the body weight in the ovariectomised rat was similar to that of postmenopausal women. There was an equal increase in the body weight in the fresh juice treated groups when compared to the other treatment. 17*β*-Estradiol reduces the sympathetic outflow in ovariectomised rats showing the role of sympathetic nervous system in controlling bone metabolism [48].

The long-term consequence of ovariectomy in mature rats is the decrease in the mineral content in the bones which was observed in the study. The ovariectomised group showed a decrease in the ash values as well as the calcium content. Treatment of the ovariectomised group with the fresh juice mixtures prevented the reduction of the calcium content as well as the ash values showing its usefulness in the prevention of bone loss.

The decrease in the femur length and weight might be due to the loss of minerals. This loss of minerals might be due to the small stimulatory effect of growth hormone on longitudinal growth through its effect on the pituitary gland [23]. The length and weight of the femur bone were increased in the other treatment groups. There was a significant increase in the length and weight in the fresh juice treated group which might be due to the deposition of the calcium.

The maximum mechanical force needed to break the femur was much less in the ovariectomised CMC treated group when compared to the other treatment groups. However the force required to break the femur bone in the other treatment groups such as estradiol, fresh juice treatment, and the extract treatment was found to be higher. The results indicate the strengthening of the bone as well as the decrease in bone loss in the osteoporotic condition.

The bone turnover was assessed by serum ALP [50] and TRAP [51–55]. These are the commonly used bone remodeling markers. ALP is for bone formation and TRAP is for bone resorption. The process of resorption and formation is always coupled and this coupling is termed as bone remodeling or bone turnover. Serum ALP was increased in the CMC treated group which might be due to the compensatory mechanism where the osteoblast is increased to compensate bone loss. Similarly there was a significant increase in the treatment groups, which points out the facilitatory effect of the treatment preparations on bone formation.

Similarly TRAP level was increased in the CMC treated groups. The treatment groups saw a decrease in the TRAP levels which might be due to the repressive effect on the osteoclast activity. Similarly the increase in the serum calcium clearly shows the increase in bone resorption. Generally calcium is maintained by negative feedback mechanism, but in our study it is noted that there might be disturbances in the mineral equilibrium which might be the reason for calcium increase.

Microscopic examination of the sections of the femur bone exhibited disruptive and lytic changes and fibrocartilaginous matrix with osteodystrophy. Groups 2, 3, and 4 showed significant restorative progress with mineralization along with fairly well-distributed osteocytes. Uniform trabeculae with variable dense matrix and shaft size were observed in group 4. The third group which was given fresh juice mixtures showed almost complete recovery with essential features of a normal bone. Histomorphological parameters were within limits and complete formation of trabeculae was observed.

*Actinidia deliciosa *(kiwi) is a natural fruit with a long history of use in humans with no serious side effects. Similarly, mangostana has also become a dietary supplement for its health promoting properties. The mangosteen fruit is unique in that it is the most abundant source of a diverse natural chemical library of xanthones and it is largely responsible for their health promoting properties.* Actinidia deliciosa *is a natural source of carotenoids, such as provitamin A beta-carotene, lutein, and zeaxanthin. The fruits are literally high in vitamin C, along with vitamins A and E, plus a considerable amount of potassium [7].

*Garcinia mangostana *is one of the popular tropical fruits. It comprises an impressive list of essential nutrients, which are required for normal growth and development and overall nutritional well-being. The fruit is rich in vitamin C and provides about 12% of RDA per 100 g. Vitamin C is a powerful water soluble antioxidant [10]. Consumption of fruits rich in vitamin C helps the body develop resistance against flu-like infectious agents and scavenging harmful, proinflammatory free radicals. The fruits are also a moderate source of B-complex vitamins such as thiamine, niacin, and folates. These vitamins act as cofactors and help the body metabolize carbohydrates, protein, and fats.

The preliminary phytochemical evaluation of the hydroalcoholic extract of the pericarp extract of* Garcinia mangostana *showed the presence of flavonoids and sterols. Earlier studies have shown that natural flavones and sterols have been helpful in bone remodeling. After consumption of these phytoestrogens and isoflavone precursors, metabolic conversions occur in the gastrointestinal tract resulting in the formation of heterocyclic phenols that are similar to the structure of estrogen. As steroidal estrogen is used in preventing osteoporosis, phytoestrogens may have a protective effect in the postmenopausal women.

The consumption of these two fruits* Actinidia deliciosa *and* Garcinia mangostana *in the treatment of osteoporosis not only encounters orthopedics but also alleviates symptoms such as antifungal [9], antioxidant, antiviral, anti-inflammatory [56], acute burns, colon cancer [57], and gastric ulcers reported in the previous study. The use of* Actinidia deliciosa *and* Garcinia mangostana *has appeared to be safe for long-term use and there have not been any reports of their short- and long-term toxicity. From these reports we conclude that the fresh juice mixture of* Actinidia deliciosa *and* Garcinia mangostana *is more potent in protecting the animals from osteoporosis than the pericarp extract of* Garcinia mangostana. *All of their effects observed in this study are similar to that of estrogen treatment. Hence the use of fresh juice mixtures of* Actinidia deliciosa *and* Garcinia mangostana *shall be considered safe and effective in the treatment of postmenopausal osteoporosis.

## 5. Conclusion

In this study, we systematically evaluated the protective effect of fresh juice* Actinidia deliciosa *and* Garcinia mangostana *on the ovariectomy-induced bone loss in female rats and explored the possible mechanism of their therapeutic effects. The data showed that daily oral administration of the fresh juice mixtures of* Actinidia deliciosa *and* Garcinia mangostana *in a three-month-old female ovariectomised rat prevented the estrogen deficiency bone loss. The combined effect of the fresh juice mixtures of* Actinidia deliciosa *and* Garcinia mangostana *was found to be a better treatment for postmenopausal osteoporosis when compared to the pericarp extract of* Garcinia mangostana*. The fresh juice mixtures can be prepared easily and conveniently and are cost-effective. Therefore the fresh juice mixtures can be used as a remedy for the treatment as well as a prophylactic of postmenopausal osteoporosis.

## Figures and Tables

**Table 1:** Body weight changes (g).

Treatment	Week 1			Week 2			Week 3			Week 4			Week 5		
	Median	25%	75%	Median	25%	75%	Median	25%	75%	Median	25%	75%	Median	25%	75%
Ovariectomised + CMC	107.000	89.000	123.750	121.500	116.000	130.750	131.000	125.250	134.750	129.000	119.500	136.000	137.000	131.500	143.500

Ovariectomised + 17*β* estradiol	112.000	101.000	131.000	127.000	123.750	132.250	130.000	126.500	132.000	130.000	129.750	133.750	140.000	131.750	144.500

Ovariectomised + fresh juice (AD + GM)	127.000	107.250	136.000	135.500	127.500	139.250	139.500	138.250	142.000	139.500	138.750	145.000	152.500	145.250	158.500

Ovariectomised + pericarp extract of *Garcinia mangostana*	118.000	103.000	139.750	129.000	122.000	130.000	129.000	118.750	132.250	135.000	129.750	136.750	141.000	135.250	142.750

**Table 2:** Ash weight (g).

Groups	Median	25%	75%
Ovariectomised + CMC	0.165	0.148	0.190
Ovariectomised + 17*β* estradiol	0.265	0.223	0.295
Ovariectomised + fresh juice (AD + GM)	0.355	0.340	0.390
Ovariectomised + pericarp extract of *Garcinia mangostana*	0.249	0.228	0.253

The difference in the median values among the treatment groups is greater than what would be expected by chance; there is a statistically significant difference (*P* < 0.001).

**Table 3:** Ash percentage (%).

Groups	Median	25%	75%
Ovariectomised + CMC	40.395	37.852	41.140
Ovariectomised + 17*β* estradiol	44.045	40.922	45.153
Ovariectomised + fresh juice (AD + GM)	52.385	51.063	53.012
Ovariectomised + pericarp extract of *Garcinia mangostana*	42.990	41.805	43.222

The difference in the median values among the treatment groups is greater than what would be expected by chance; there is a statistically significant difference (*P* < 0.001).

**Table 4:** Measurement of length femur bone (mm).

Groups	Median	25%	75%
Ovariectomised + CMC	2.085	2.005	2.235
Ovariectomised + 17*β* estradiol	3.420	3.295	3.555
Ovariectomised + fresh juice mixtures (AD + GM)	3.640	3.518	3.700
Ovariectomised + pericarp extract of *Garcinia mangostana *	3.375	3.297	3.410

The difference in the median values among the treatment groups is greater than what would be expected by chance; there is a statistically significant difference (*P* < 0.001).

**Table 5:** Measurement of weight of femur bone (g).

Groups	Median	25%	75%
Ovariectomised + CMC	0.310	0.285	0.335
Ovariectomised + 17*β* estradiol	0.510	0.472	0.552
Ovariectomised + fresh juice mixtures (AD + GM)	0.575	0.548	0.615
Ovariectomised + pericarp extract of *Garcinia mangostana *	0.455	0.408	0.505

The difference in the median values among the treatment groups is greater than what would be expected by chance; there is a statistically significant difference (*P* < 0.001).

**Table 6:** Three-point bending test (N).

Groups	Median	25%	75%
Ovariectomised + CMC	13.875	13.085	14.140
Ovariectomised + 17*β* estradiol	43.435	37.425	46.535
Ovariectomised + fresh juice mixtures (AD + GM)	74.220	66.983	79.017
Ovariectomised + pericarp extract of *Garcinia mangostana *	41.4555	39.780	48.573

The difference in the median values among the treatment groups is greater than what would be expected by chance; there is a statistically significant difference (*P* < 0.001).

**Table 7:** Serum biochemical estimation, TRAP.

Groups	Median	25%	75%
Ovariectomised + CMC	8.782	8.782	8.822
Ovariectomised + 17*β* estradiol	5.290	5.290	5.395
Ovariectomised + fresh juice mixtures (AD + GM)	4.780	4.780	4.923
Ovariectomised + pericarp extract of *Garcinia mangostana *	5.473	5.473	5.645

The difference in the median values among the treatment groups is greater than what would be expected by chance; there is a statistically significant difference (*P* < 0.001).

**Table 8:** Serum biochemical estimation, ALP.

Groups	Median	25%	75%
Ovariectomised + CMC	545.000	539.750	550.750
Ovariectomised + 17*β* estradiol	264.000	263.000	265.250
Ovariectomised + fresh juice mixtures (AD + GM)	482.500	460.000	485.000
Ovariectomised + pericarp extract of *Garcinia mangostana *	233.000	211.250	247.250

The difference in the median values among the treatment groups is greater than what would be expected by chance; there is a statistically significant difference (*P* < 0.001).

**Table 9:** Serum biochemical estimation, calcium.

Groups	Median	25%	75%
Ovariectomised + CMC	54.795	52.460	56.313
Ovariectomised + 17*β* estradiol	73.235	72.670	74.240
Ovariectomised + fresh juice mixtures (AD + GM)	79.550	78.400	80.160
Ovariectomised + pericarp extract of *Garcinia mangostana *	61.990	60.178	63.010

The difference in the median values among the treatment groups is greater than what would be expected by chance; there is a statistically significant difference (*P* < 0.001).

## Abbreviations

OVX: Ovariectomy/ovariectomised
CPCSEA: Committee for the Purpose of Control and Supervision of Experiments on Animals
IAEC: Institutional Animal Ethical Committee
ALP: Alkaline phosphatase
TRAP: Tartrate resistant acid phosphate
SEM: Standard error of mean
ANOVA: Analysis of variance
CMC: Carboxymethyl cellulose
AD: * Actinidia deliciosa*
GM: * Garcinia mangostana*
FDA: Food and Drug Administration.
